# Temporal Trends in Cardiometabolic Control Among Canadian Adults: A Comparative Analysis of Hypertension and Diabetes Using the Canadian Health Measures Survey (CHMS) 2008–2019

**DOI:** 10.7759/cureus.97547

**Published:** 2025-11-23

**Authors:** Adaora W Mochu, Omotolani A Meseko, Osasuyi A Emovon, Chibuzo N Nwodo, Ifesinachi Nwankwor, Afolake A Adebayo, Omotayo O Amure, Manuel B Posadas, Lebechi N Opara

**Affiliations:** 1 Family Medicine, University of Perpetual Help System DALTA, JONELTA Foundation School of Medicine, Las Piñas, PHL; 2 Faculty of Medicine, Tbilisi State Medical University, Tbilisi, GEO; 3 Oral and Maxillofacial Surgery, Obafemi Awolowo University Teaching Hospital, Ife, NGA; 4 Otorhinolaryngology, Royal Derby Hospital, Derby, GBR; 5 General Practice, General Hospital Agulu, Agulu, NGA; 6 Family Medicine, Nnamdi Azikiwe University, Nnewi, NGA; 7 Family Medicine, Lagos State University Teaching Hospital, Ikeja, NGA; 8 Medicine, University of the East Ramon Magsaysay Memorial Medical Center, Quezon City, PHL; 9 Obstetrics and Gynaecology, General Hospital Isolo, Isolo, NGA

**Keywords:** canada, cardiometabolic health, chms, diabetes, hypertension, temporal trends

## Abstract

Background: Hypertension and diabetes remain major contributors to cardiovascular morbidity and mortality in Canada. Monitoring their trends is critical to evaluating public health progress in prevention and disease management.

Objective: To descriptively summarize published Canadian Health Measures Survey (CHMS) estimates on the prevalence, awareness, treatment, and control of hypertension and diabetes among Canadian adults aged 20-79 years across the 2008-2019 survey cycles.

Methods: Descriptive analysis was conducted using aggregated CHMS combined-cycle data (2008-2011, 2012-2015, and 2016-2019). Weighted proportions and absolute counts published by Statistics Canada were organized and analyzed in Stata version 18. Data were stratified by sex and age group, and graphical visualization was applied to highlight temporal and demographic patterns.

Results: Hypertension prevalence remained stable over time, accompanied by modest improvements in treatment and control rates. Conversely, diabetes prevalence continued to rise, with limited gains in glycemic control. These patterns suggest differential progress in managing cardiometabolic conditions among Canadian adults.

Conclusion: National strategies appear to have improved hypertension outcomes but less effectively addressed diabetes control. Strengthening preventive interventions, enhancing primary care integration, and improving access to chronic disease management resources remain essential for advancing cardiometabolic health in Canada.

## Introduction

In Canada, increased rates of cardiometabolic risk factors coupled with an increasing age of the population have resulted in hypertension and diabetes being declared public-health concerns in terms of detection, treatment, and management [1]. These are significant public health problems, as they are so widespread, long-term, and associated with preventable issues such as heart attack, kidney failure, and stroke [2]. The level of control is low in most groups despite the fact that evidence-based guidelines and universal health care are implemented [3]. The trends in disease rates and control over time will allow us to assess the effectiveness of national health programs and identify the gaps in health disparities. 

The prevalence of hypertension and diabetes has changed over the past decade, as documented in national surveillance reports and prior epidemiologic studies examining cardiometabolic trends in Canada [4]. Hypertension affects approximately one in five adults, while diabetes impacts nearly one in ten Canadians, both conditions sharing overlapping determinants such as obesity, aging, and physical inactivity [5,6]. These diseases often coexist, compounding cardiovascular risk and straining health system resources through increased rates of hospitalization, medication use, and long-term management costs [7]. Understanding temporal changes in disease prevalence, treatment, and control provides important background surveillance information that helps contextualize national efforts to improve cardiometabolic health [8,9].

Between 2007 and 2019, the age-standardized prevalence of hypertension remained relatively stable at approximately 21.7% to 24.2%, while diabetes prevalence among adults with comorbidities fluctuated over time, ranging from 22.2% to 27.4% across survey periods [10]. Hypertension awareness was 78.9% during 2007-2017 but has risen to 83.5% in 2018-2019 [11]. Most hypertension is managed in primary care; therefore, improving hypertension care at the population level necessitates prioritizing primary care [12]. These differences roughly suggest greater success in dealing with hypertension than diabetes [13]. The improvement in hypertension control was attributed to increased use of combination therapy and increased dissemination of guidelines among primary care providers [14]. Conversely, diabetes management was faced with constant barriers such as poor adherence to medication, lifestyle issues, and unequal access to specialized care [15].

The main objective of the study is to examine temporal trends in the prevalence and control of hypertension and diabetes among Canadian adults aged 20-79 years between 2008 and 2019 using data from the Canadian Health Measures Survey (CHMS) [16,17]. From 2008 to 2019, hypertension prevalence levels in Canada remained stable, and control rates improved; hence, the prevalence of diabetes rose, and glycemic control plateaued. The data highlight the importance of targeting national strategies to improve chronic disease management, improve equitable access to care, and improve monitoring of trends in cardiometabolic health outcomes through robust surveillance systems.

## Materials and methods

Study design and data source

This study employed a repeated cross-sectional design using data from the Canadian Health Measures Survey (CHMS), a nationally representative, population-based survey conducted by Statistics Canada [16,17]. The CHMS collects extensive health, clinical, and laboratory information from Canadians through household interviews and physical examinations conducted in mobile examination centers. Data were drawn from six CHMS cycles conducted between 2007 and 2019, which were pooled into three combined periods (2008-2011, 2012-2015, and 2016-2019) to ensure sufficient sample sizes and enhance the reliability of estimates. These data represent the non-institutionalized Canadian population living in the 10 provinces, excluding individuals residing in the territories, on reserves, in institutions, or in remote regions. 

Study population

The analytic sample included adults aged 20 to 79 years who participated in the CHMS and had valid measurements of blood pressure, blood glucose, and glycated hemoglobin (HbA1c). Pregnant women and participants with missing or invalid laboratory data were excluded, consistent with Statistics Canada’s analytical standards for these health indicators. This age range was selected to maintain comparability with published CHMS summary tables and to align with the age group most affected by cardiometabolic conditions. 

Variables and measures

The primary outcomes of interest were the prevalence, awareness, treatment, and control of hypertension and diabetes among Canadian adults. For both conditions, definitions followed Statistics Canada’s established criteria. Individuals were classified as having diabetes if they self-reported a physician diagnosis of diabetes, reported using blood glucose-lowering medication in the past month, or had an HbA1c level ≥6.5%. Diabetes awareness referred to participants who reported being told by a health professional that they had diabetes or who reported active use of blood glucose-lowering medication. Treatment was defined as self-reported medication use for diabetes management, while control denoted individuals with diabetes who achieved an HbA1c <7.0%. Prediabetes was defined as HbA1c between 6.0% and 6.4% among individuals without self-reported diabetes or medication use.

Hypertension was defined as a mean systolic blood pressure ≥140 mmHg, diastolic blood pressure ≥90 mmHg, or current use of antihypertensive medication. Awareness of hypertension indicated a self-reported prior diagnosis or current antihypertensive medication use. Treatment referred to the use of antihypertensive medications, while control was defined as treated individuals with measured blood pressure <140/90 mmHg. All definitions were consistent with CHMS technical documentation and national surveillance standards to allow comparability across cycles and with previously published studies.

Statistical analysis

Descriptive analyses were conducted to summarize diabetes and hypertension indicators among adults aged 20-79 years using data from the Canadian Health Measures Survey (CHMS) combined cycles. Weighted proportions (percent) and absolute counts (number of persons) were used to align with Statistics Canada’s reporting framework.

Data were stratified by sex (male and female) and age group (20-79 years) to facilitate cycle-by-cycle descriptive comparisons in prevalence, awareness, treatment, and control across the survey periods (2008-2011, 2012-2015, and 2016-2019). Quality markers provided within the CHMS (“E” for estimates to be used with caution and “F” for estimates too unreliable to be published) were retained in accordance with established methodological standards.

Because the study relied on published aggregated CHMS summary tables rather than individual-level microdata, the estimates represent descriptive proportions only. Confidence intervals, age standardization, and survey-weighted variance estimation could not be applied. As a result, the figures and tables illustrate cycle-to-cycle patterns, and the analysis does not assess whether observed differences exceed sampling variability. These comparisons should therefore be interpreted as descriptive patterns, not inferentially confirmed temporal trends.

All data management and descriptive analyses, including the generation of graphs, were performed using Stata Version 18 (StataCorp LLC, College Station, TX). The graphical summaries were used solely to visualize the published CHMS estimates and to highlight broad patterns across demographic groups.

Ethical considerations

The CHMS protocol received ethical approval from Health Canada’s Research Ethics Board, and informed consent was obtained from all participants prior to data collection. The present analysis used anonymized, publicly available data accessed in accordance with Statistics Canada’s data use policies. No additional ethical approval was required for secondary data analysis. All analyses adhered to the confidentiality and privacy standards set forth by Statistics Canada to ensure that individual respondents could not be identified directly or indirectly.

## Results

**Figure 1:**
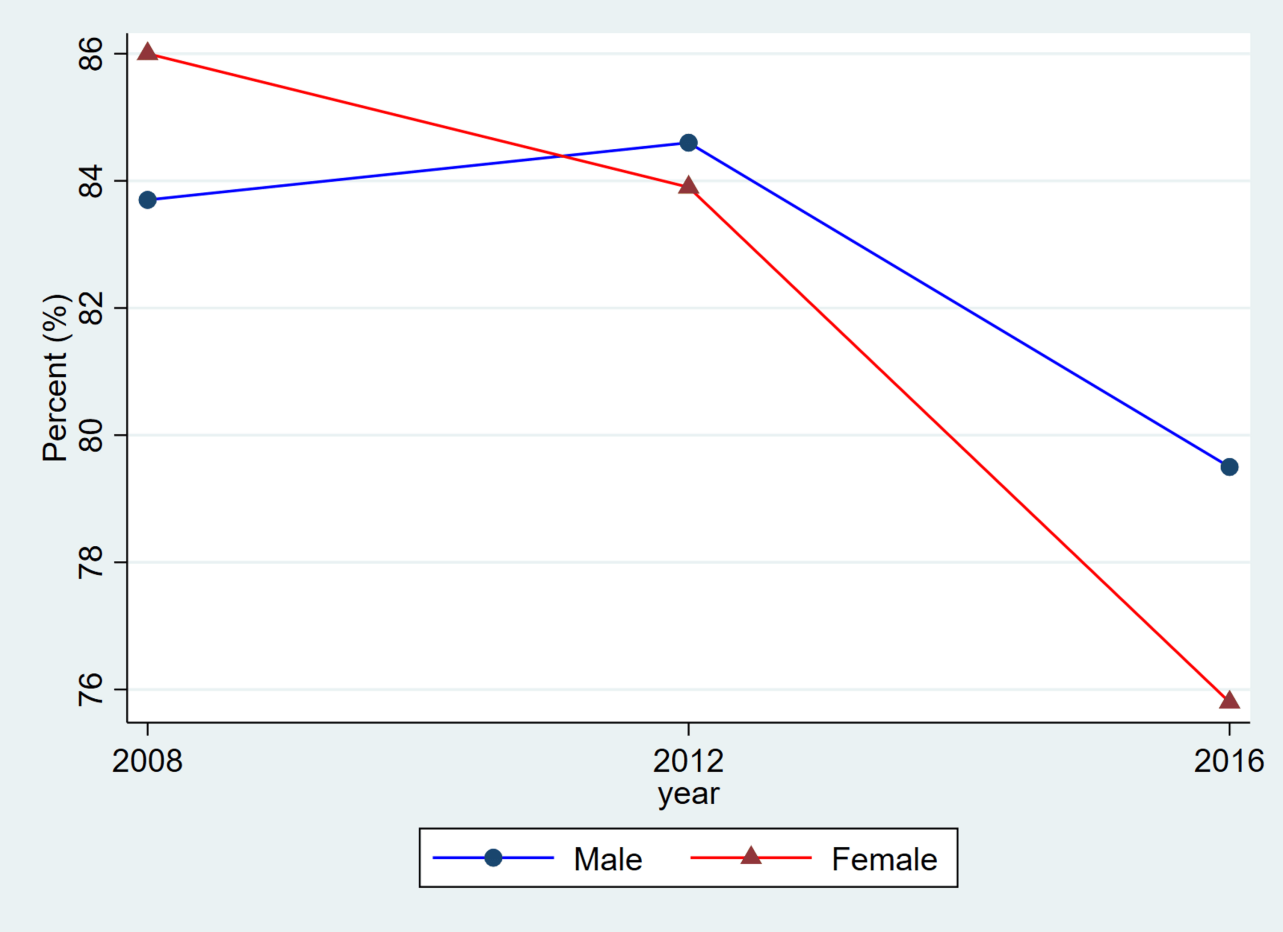
Trends in awareness of hypertension by sex, ages 20–79, 2008–2019 This figure represents the percent of adults with hypertension who reported being aware of their condition. Blue lines and circles indicate males, while red lines and triangles indicate females. Data reflect weighted estimates published by Statistics Canada.

**Figure 2:**
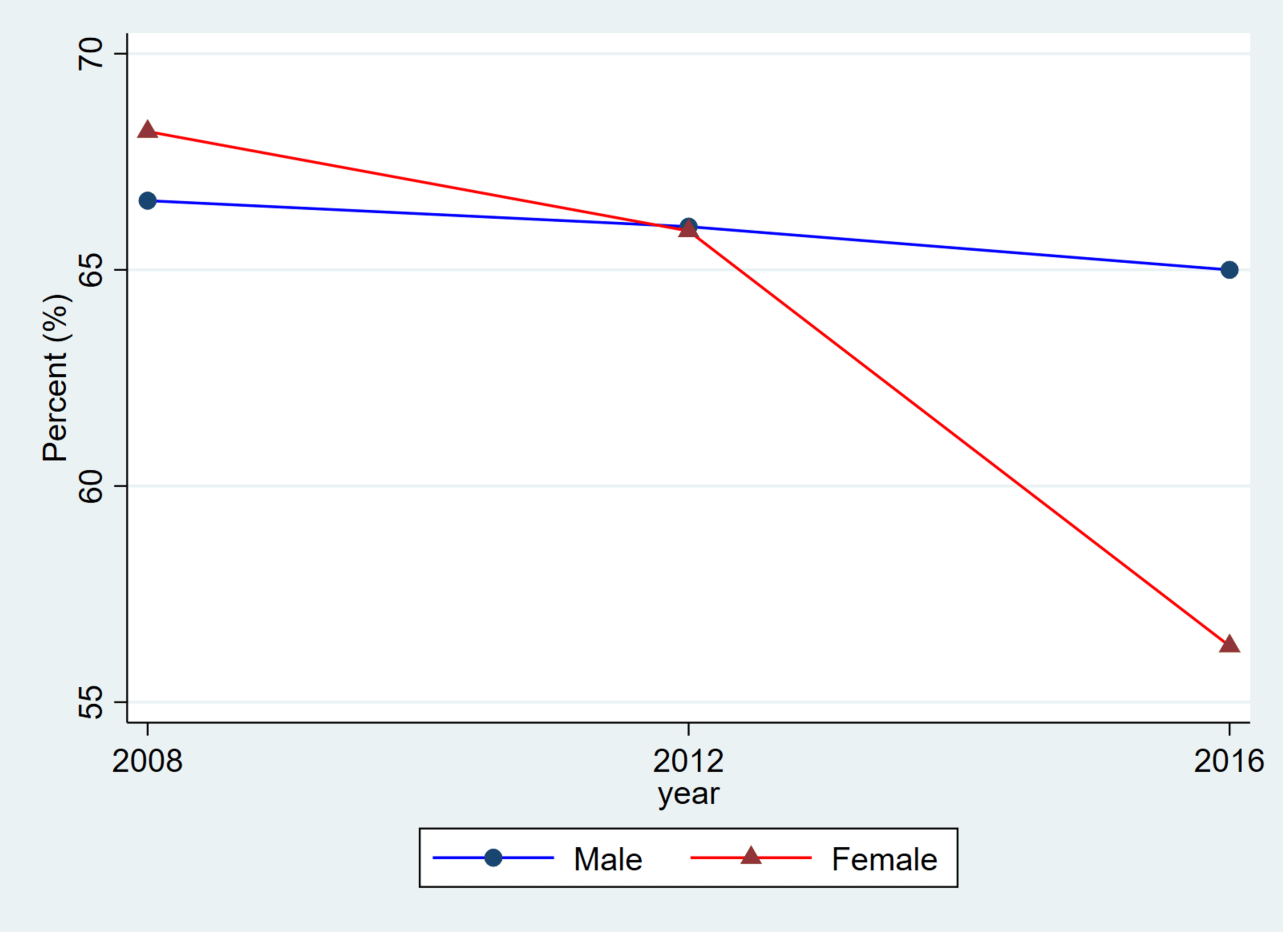
Trends in blood pressure control among adults with hypertension by sex, ages 20–79, 2008–2019 This figure represents the percent of adults with hypertension whose blood pressure was controlled (systolic <140 mmHg and diastolic <90 mmHg). Blue lines and circles indicate males, while red lines and triangles indicate females. Data reflect weighted estimates published by Statistics Canada.

**Figure 3:**
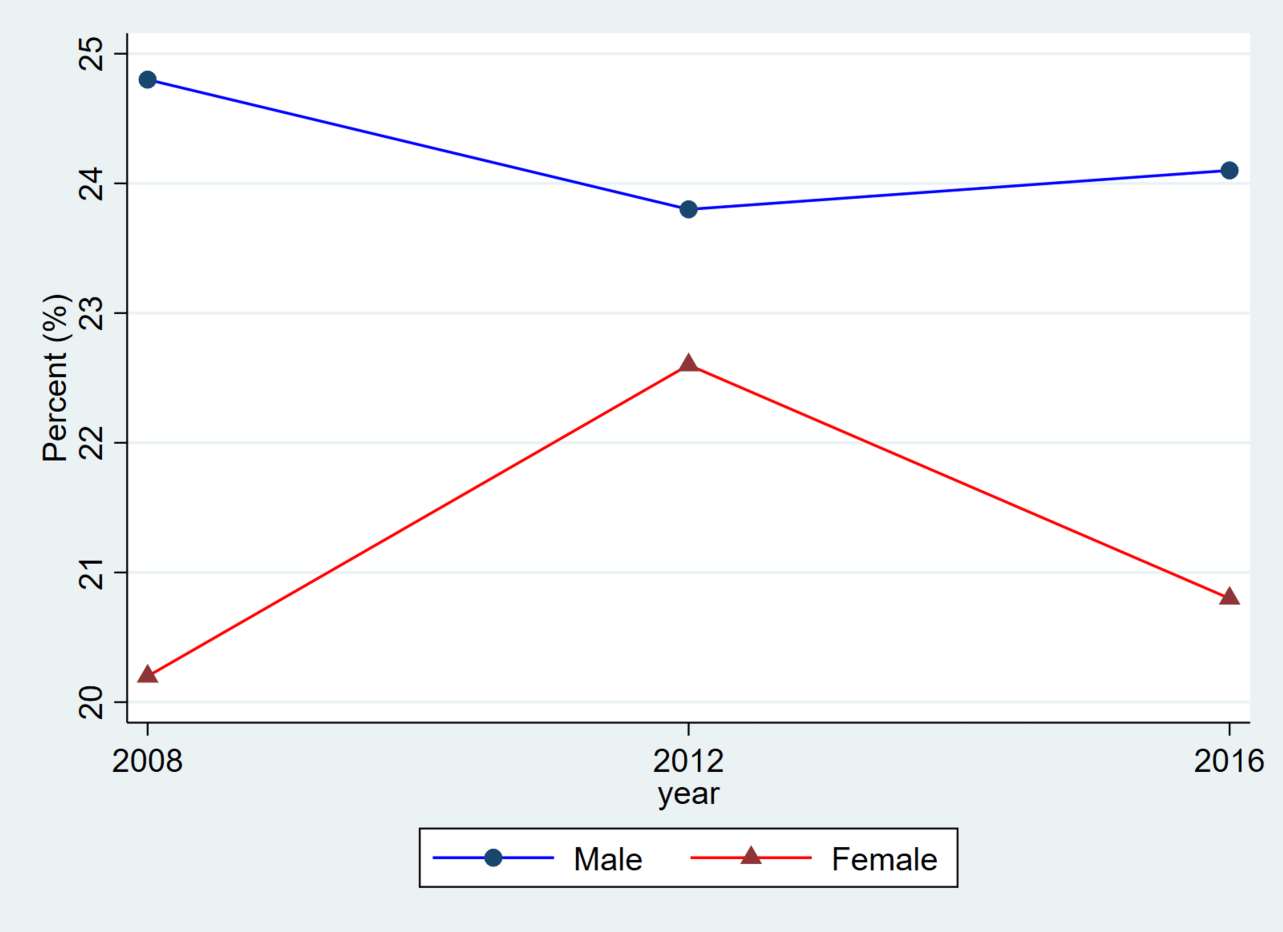
Trends in hypertension prevalence by sex, ages 20–79, 2008–2019 This figure represents the percent of adults meeting diagnostic criteria for hypertension (systolic ≥140 mmHg or diastolic ≥90 mmHg, or use of antihypertensive medication). Blue lines and circles indicate males, while red lines and triangles indicate females. Data reflect weighted estimates published by Statistics Canada.

**Figure 4:**
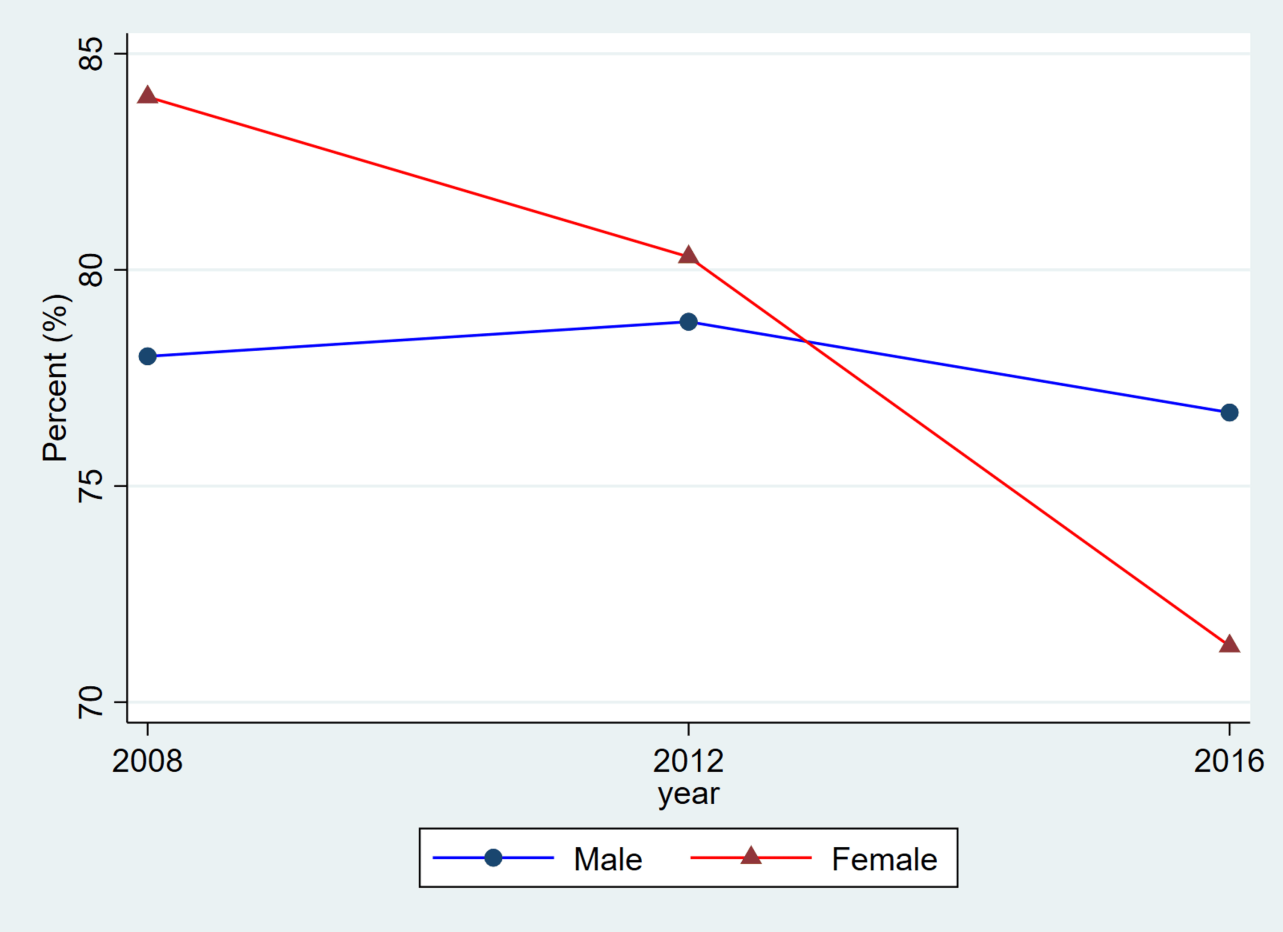
Trends in hypertension treatment by sex, ages 20–79, 2008–2019 This figure represents the percent of adults with hypertension who reported using antihypertensive medication. Blue lines and circles indicate males, while red lines and triangles indicate females. Data reflect weighted estimates published by Statistics Canada.

Table 1 and Figures 1-4 present cycle-by-cycle descriptive comparisons of hypertension prevalence, awareness, treatment, and control among Canadian adults across three combined CHMS periods (2008-2011, 2012-2015, and 2016-2019). These values reflect published CHMS summary estimates and therefore represent descriptive proportions rather than inferentially tested trends. The comparisons provide an overview of how these indicators varied across survey cycles. Examining these indicators across time provides insight into Canada’s progress in detecting, managing, and controlling hypertension in the population and highlights potential differences between males and females in disease burden and management outcomes.

**Table 1:** Aggregated CHMS estimates of hypertension prevalence, awareness, treatment, and control among adults aged 20–79 years, by sex, Canada (2008–2019) These values are aggregated estimates obtained directly from published Statistics Canada CHMS summary tables. They represent weighted proportions and counts without accompanying confidence intervals, unweighted sample sizes, or survey-weighted variance estimates. No variance estimation or inferential statistical testing was performed. Estimates flagged with CHMS quality indicators (“E” and “F”) follow Statistics Canada’s reporting standards and should be interpreted with caution.

-	FEMALE			MALE		
Measure	2008–2011 N (%)	2012–2015 N (%)	2016–2019 N (%)	2008–2011 N (%)	2012–2015 N (%)	2016–2019 N (%)
Hypertension prevalence	2,416,500 (20.2)	2,858,300 (22.6)	2,722,000 (20.8)	2,961,500 (24.8)	3,007,400 (23.8)	3,180,200 (24.1)
Awareness	2,078,000 (86.0)	2,397,700 (83.9)	2,063,500 (75.8)	2,479,500 (83.7)	2,543,900 (84.6)	2,528,400 (79.5)
Treatment	2,030,200 (84.0)	2,295,700 (80.3)	1,939,500 (71.3)	2,310,000 (78.0)	2,369,900 (78.8)	2,438,600 (76.7)
Control	1,648,000 (68.2)	1,884,400 (65.9)	1,531,500 (56.3)	1,971,400 (66.6)	1,986,100 (66.0)	2,068,600 (65.0)

The findings from Table 1 reveal that between 2008 and 2019, hypertension remained a prevalent chronic condition among Canadian adults, with consistently higher rates observed in males compared to females. Among females, prevalence rose from 2,416,500 (20.2%) in 2008-2011 to 2,858,300 (22.6%) in 2012-2015 before slightly declining to 2,722,000 (20.8%) in 2016-2019. Among males, corresponding estimates were higher at 2,961,500 (24.8%), 3,007,400 (23.8%), and 3,180,200 (24.1%), indicating a relatively stable prevalence over time. Awareness of hypertension was generally high for both sexes, though a declining trend was noted in recent years. Female awareness decreased from 2,078,000 (86.0%) to 2,063,500 (75.8%) across the study period, while male awareness showed a modest drop from 2,479,500 (83.7%) to 2,528,400 (79.5%).

Treatment coverage, as reflected in the published CHMS summary estimates, shows lower reported proportions for women in the most recent cycle and relatively similar proportions for men across cycles. Because these values are derived from aggregate CHMS tables without variance estimates, these descriptive differences cannot be interpreted as statistically tested changes. Hypertension control demonstrated a gradual decline over time, particularly among females, decreasing from 1,648,000 (68.2%) in 2008-2011 to 1,531,500 (56.3%) in 2016-2019. Among males, control levels remained higher and more stable, with 1,971,400 (66.6%) controlled in 2008-2011 and 2,068,600 (65.0%) in 2016-2019.

Overall, the patterns illustrated in Figure 1 are consistent with the descriptive values shown in Table 1. Both men and women exhibited relatively stable prevalence estimates across cycles, while awareness, treatment, and control showed modest fluctuations. As these estimates are drawn from CHMS summary tables and do not include variance measures, these differences should be interpreted as descriptive cycle-to-cycle patterns rather than confirmed temporal changes.

**Figure 5:**
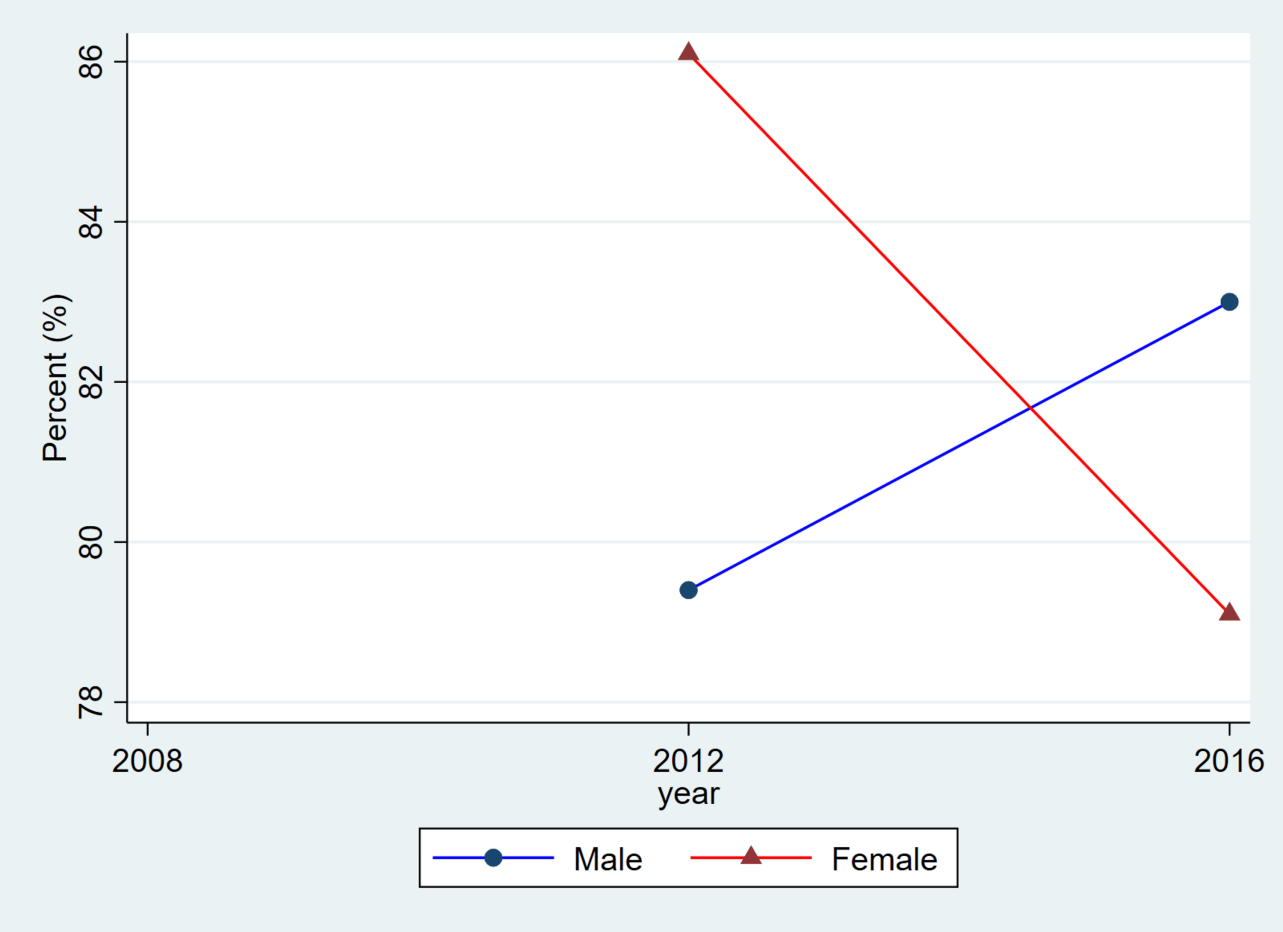
Trends in awareness of diabetes by sex, 2008–2019 This figure represents the percent of adults who were aware of their diabetes diagnosis. Blue lines and circles indicate males, while red lines and triangles indicate females. Data reflect weighted estimates published by Statistics Canada.

**Figure 6:**
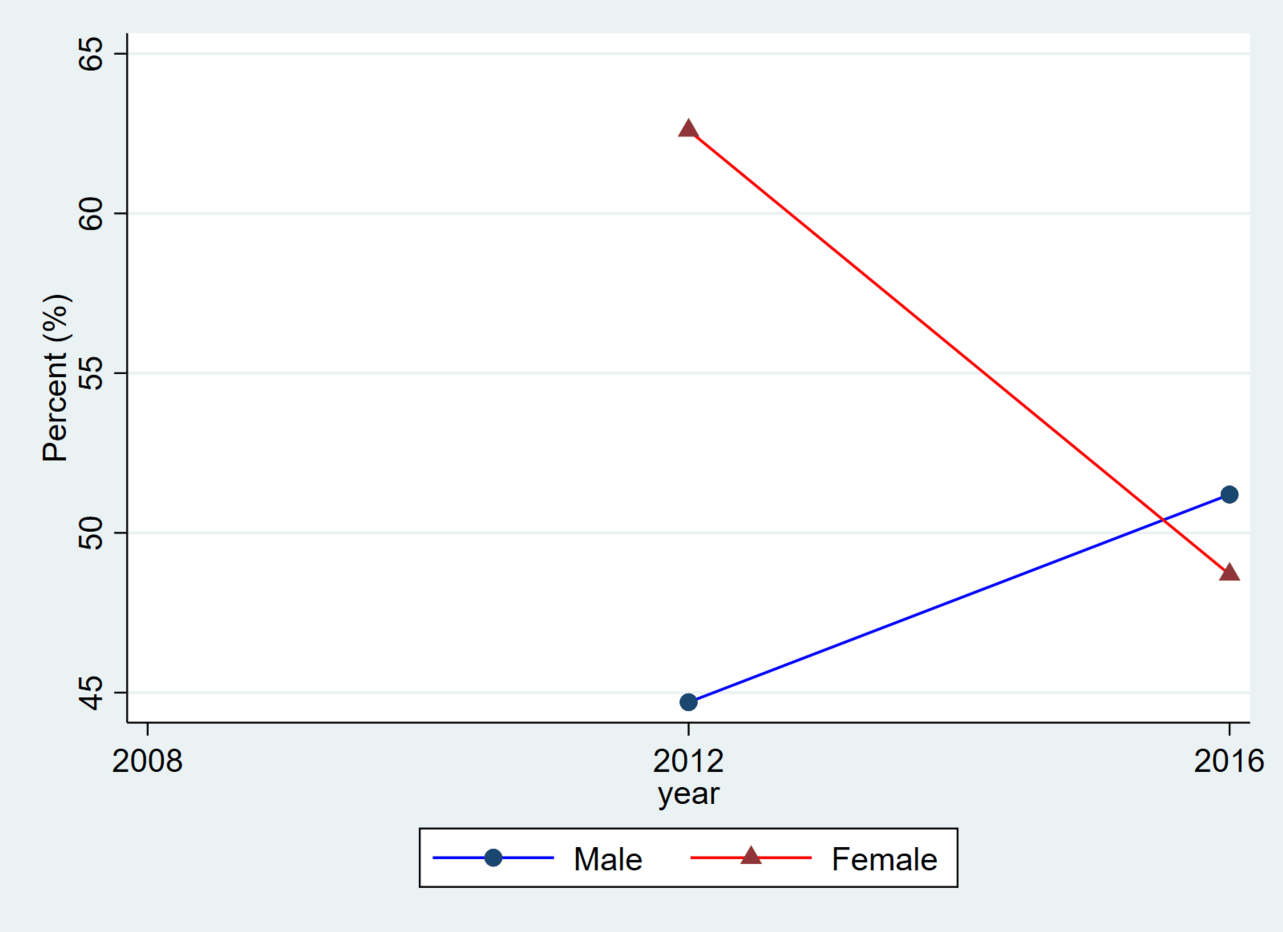
Trends in controlled blood glucose by sex, 2008–2019 This figure represents the percent of adults with diabetes whose blood glucose levels were controlled (HbA1c < 7.0%). Blue lines and circles indicate males, while red lines and triangles indicate females. Data reflect weighted estimates published by Statistics Canada.

**Figure 7:**
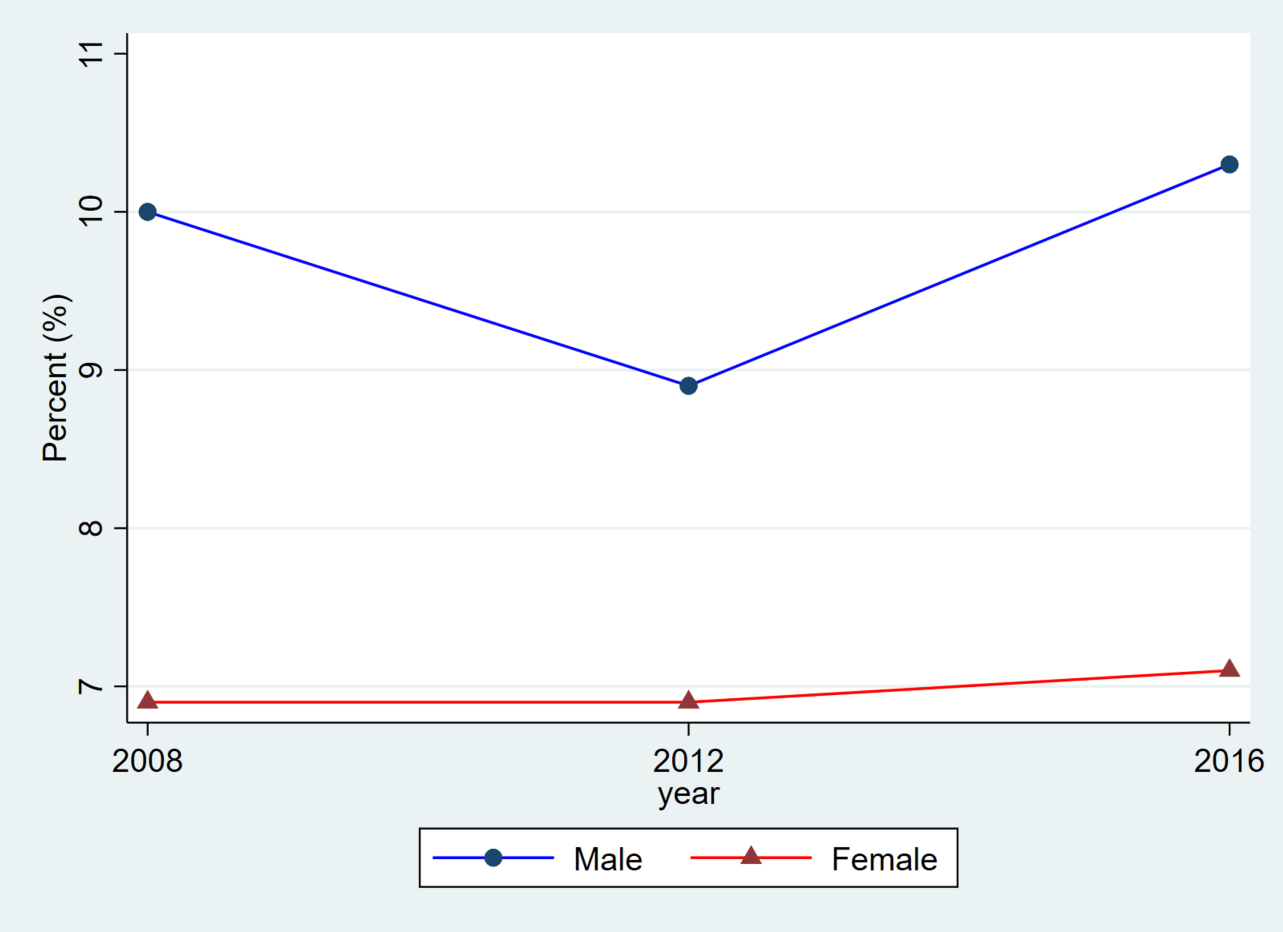
Trends in diabetes prevalence by sex, 2008–2019 This figure represents the percent of adults meeting diagnostic criteria for diabetes. Blue lines and circles indicate males, while red lines and triangles indicate females. Data reflect weighted estimates published by Statistics Canada.

**Figure 8:**
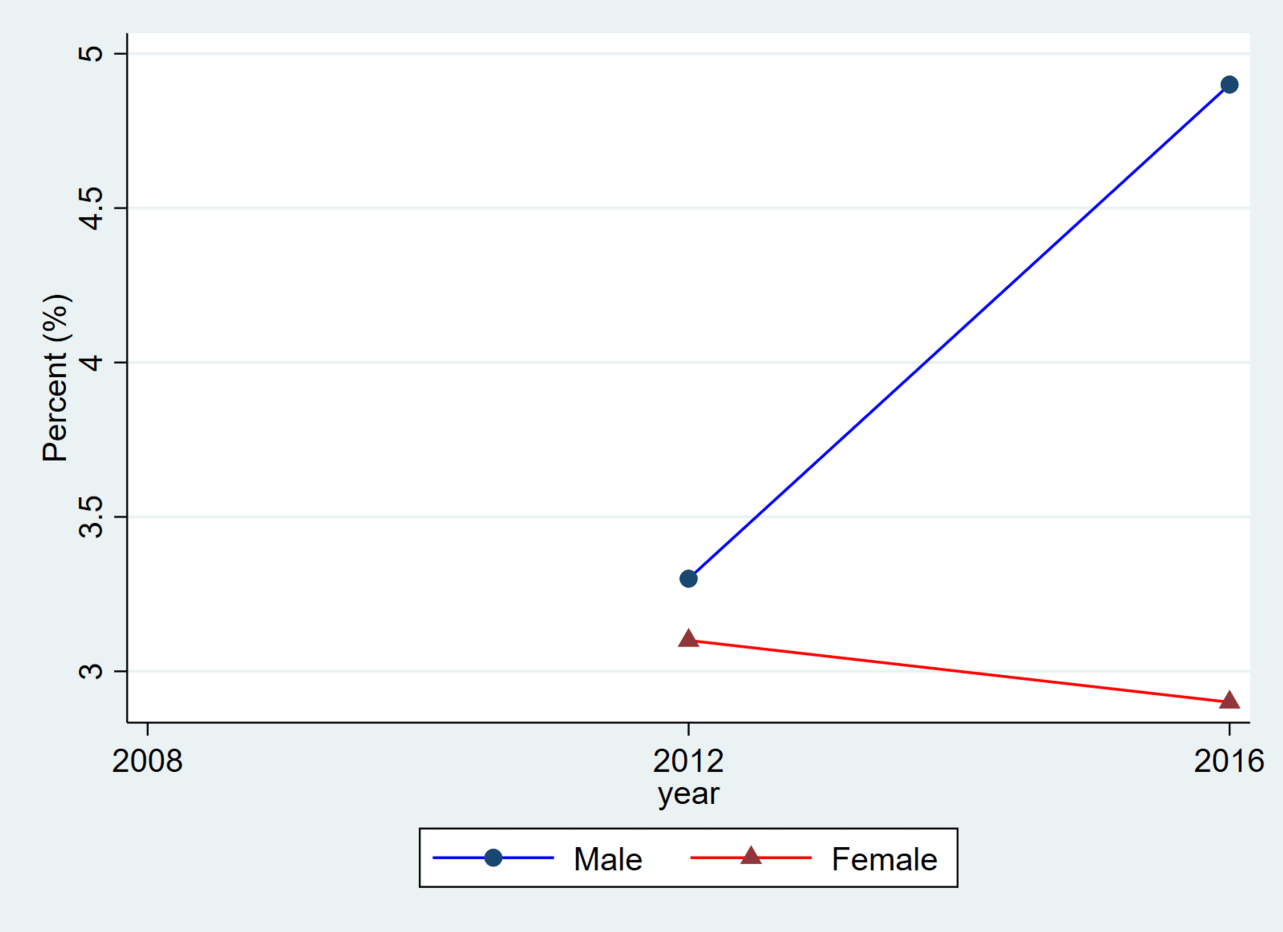
Trends in prediabetes prevalence by sex, 2008–2019 This figure represents the percent of adults meeting criteria for prediabetes (fasting glucose 6.1–6.9 mmol/L or HbA1c 6.0–6.4%). Blue lines and circles indicate males, while red lines and triangles indicate females. Data reflect weighted estimates published by Statistics Canada.

**Figure 9:**
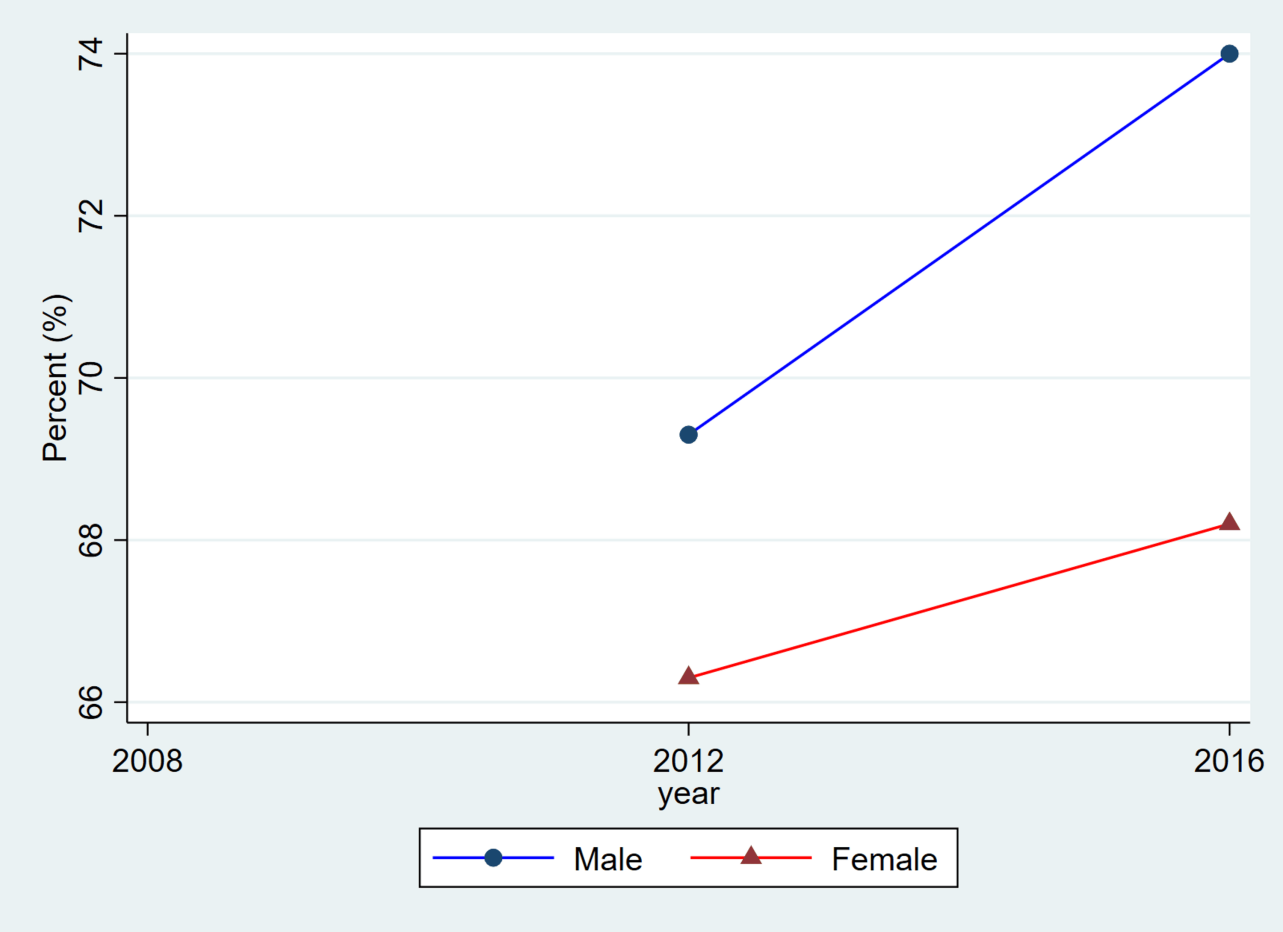
Trends in diabetes treated with medication by sex, 2008–2019 This figure represents the percent of adults with diabetes who reported using antidiabetic medication. Blue lines and circles indicate males, while red lines and triangles indicate females. Data reflect weighted estimates published by Statistics Canada.

Table 2 and Figures 5-9 summarize descriptive cycle-by-cycle comparisons of diabetes and prediabetes indicators among Canadian adults aged 20-79 years based on the combined CHMS cycles from 2008-2019. These indicators are taken directly from Statistics Canada’s publicly released CHMS combined-cycle estimates. Because the analysis relies entirely on these predetermined, aggregate indicators, no new variable construction or individual-level data processing was performed. Data are presented by sex to highlight potential differences in disease patterns and management.

**Table 2:** Aggregated CHMS estimates of diabetes prevalence, awareness, treatment, control, and prediabetes among adults aged 20–79 years, by sex, Canada (2008–2019) These values are aggregated estimates drawn directly from published Statistics Canada CHMS summary tables. The figures represent weighted proportions and counts and do not include confidence intervals, unweighted sample sizes, or survey-weighted variance estimates. No variance estimation or inferential statistical testing was performed. Estimates flagged with CHMS quality indicators (“E” and “F”) follow Statistics Canada’s reporting standards and should be interpreted with caution.

-	FEMALE			MALE		
Measure	2008–2011 N (%)	2012–2015 N (%)	2016–2019 N (%)	2008–2011 N (%)	2012–2015 N (%)	2016–2019 N (%)
Prevalence of diabetes	795,800 (6.9)	818,300 (6.9)	916,600 (7.1)	1,164,600 (10.0)	1,060,900 (8.9)	1,340,900 (10.3)
Awareness	F	704,800 (86.1)	724,600 (79.1)	F	841,800 (79.4)	1,113,000 (83.0)
Treatment	F	542,300 (66.3)	625,400 (68.2)	F	735,500 (69.3)	992,500 (74.0)
Control (HbA1c < 7.0%)	F	512,100 (62.6)	446,400 (48.7)	F	474,100 (44.7)	686,700 (51.2)
Prediabetes	F	363,500 (3.1^E^)	374,500 (2.9^E^)	F	398,000 (3.3)	635,600 (4.9)

Across the 2008-2019 period, diabetes prevalence among Canadian adults showed a slight but steady increase. Among women, the number of individuals with diabetes rose from 795,800 (6.9%) in 2008-2011 to 916,600 (7.1%) in 2016-2019. Men consistently had higher prevalence rates, with estimates increasing from 1,164,600 (10.0%) to 1,340,900 (10.3%) across the same years. This pattern highlights a continuing gap between men and women, with males experiencing a greater overall burden of diabetes.

Awareness of diabetes among those diagnosed remained high but appeared to decline slightly over time, particularly among women. Female awareness decreased from 704,800 (86.1%) in 2012-2015 to 724,600 (79.1%) in 2016-2019, while men reported similar or slightly higher levels, from 841,800 (79.4%) to 1,113,000 (83.0%). Treatment rates followed a comparable pattern, showing modest improvement. Among women, treatment increased from 542,300 (66.3%) to 625,400 (68.2%), and among men, from 735,500 (69.3%) to 992,500 (74.0%). These results suggest that most individuals with diabetes remain in care and are receiving treatment, though the small fluctuations may reflect differences in disease awareness or medication adherence.

In contrast, control of blood glucose (defined as HbA1c below 7.0%) showed fewer encouraging results. Among women, control declined from 512,100 (62.6%) in 2012-2015 to 446,400 (48.7%) in 2016-2019. Among men, control improved slightly, from 474,100 (44.7%) to 686,700 (51.2%), yet still remained below optimal levels. This drop in glycemic control, despite relatively stable treatment coverage, may reflect growing challenges in long-term disease management and lifestyle modification.

Prediabetes estimates remained low overall but showed minor changes over time. Among women, prevalence was 363,500 (3.1%) in 2012-2015 and 374,500 (2.9%) in 2016-2019, while among men it rose modestly from 398,000 (3.3%) to 635,600 (4.9%). This upward shift among men could signal an increasing number of individuals at risk of developing diabetes.

Overall, the trends presented in Figures 5-9 reinforce the data summarized in Table 2. While diabetes and prediabetes prevalence appear relatively stable across cycles, the descriptive estimates show lower values for diabetes awareness and control in the most recent period, particularly among women; however, these differences cannot be interpreted as statistically meaningful because the analysis is based on aggregated CHMS data without variance estimation. These findings point to the need for continued emphasis on diabetes prevention, early detection, and consistent management to sustain progress in reducing the burden of cardiometabolic disease among Canadian adults.

## Discussion

The present analysis examined temporal trends in the prevalence, awareness, treatment, and control of hypertension and diabetes among Canadian adults aged 20-79 years using data from the Canadian Health Measures Survey (CHMS) from 2008 to 2019. Overall, findings reveal a pattern of stability in hypertension prevalence, modest gains in treatment and control, and a continuing rise in diabetes prevalence with largely stagnant glycemic control. These results highlight persistent disparities in cardiometabolic management and underscore the differing progress achieved in the control of hypertension compared with diabetes over the past decade.

Analysis across Canada has shown significant improvements in the control of hypertension but a lack of progress in the control of diabetes in Canadian adults [18]. These findings are in line with those for other areas of the world where management of hypertension has improved more rapidly as a result of simpler pharmacologic regimens and sound primary care follow-up systems [19]. Diabetes management is still complicated by the necessity of individualized lifestyle modification, self-monitoring, and insulin management, all factors that are strongly influenced by socioeconomic and behavioral determinants [20]. To promote cardiometabolic health in Canada, it needs to strengthen integrated primary and specialty care, increase medication affordability with the use of national pharm-care, and promote culturally sensitive prevention programs targeted to diverse communities [21,22].

The descriptive CHMS estimates show relatively stable hypertension prevalence across cycles and modest variation in awareness and treatment proportions. These patterns are consistent with previously published national surveillance reports that have noted similar population-level estimates [10]. However, because our analysis relies on aggregated CHMS tables without the ability to assess statistical significance or examine determinants, these descriptive patterns cannot be linked to specific clinical practices, guideline uptake, or system-level changes. The Diabetes Canada 2018 Clinical Practice Guidelines emphasize individualized blood-pressure targets, regular monitoring, and first-line therapies such as ACE inhibitors or ARBs for patients with diabetes, particularly those with albuminuria or cardiovascular risk factors [3,10,12]. Effective blood-pressure control in individuals with diabetes markedly reduces the risk of both microvascular and macrovascular complications. Yet, evidence from Canadian primary care shows that only about 40-49% of adults achieved target blood-pressure levels by 2020, with younger adults and women particularly affected [14]. These findings suggest that improving hypertension management within diabetes care remains a crucial step toward comprehensive cardiovascular risk reduction.

The persistent rise in diabetes prevalence and limited progress in glycemic control mirror both national and international patterns [18-20]. Despite high awareness and treatment rates, control remains suboptimal, especially among men, reflecting the complex behavioral, socioeconomic, and healthcare-access factors that shape self-management and adherence [15,20]. Unlike hypertension, diabetes management relies heavily on individualized monitoring, medication adherence, and lifestyle modification, all of which are influenced by environmental and social conditions. Socioeconomic inequalities continue to shape cardiometabolic outcomes across Canada, where the prevalence of hypertension, diabetes, smoking, alcohol use, and obesity varies widely by income, education, and immigration status [1,4,20]. Immigrants and individuals with lower socioeconomic status often face barriers such as unemployment, language differences, and reduced access to culturally sensitive care, contributing to worse outcomes. Conversely, higher income and education levels are associated with better access to care and improved disease control [4,20]. 

To prevent cardiometabolic disorders from getting worse, it's important to catch key warning signs early, such as high blood pressure, abnormal cholesterol levels, poor blood sugar control, and lack of physical activity. Acting quickly on these warning signs can slow the disease's progression. Researchers found that from 1990 to 2019, high blood pressure levels stayed relatively stable across various demographic groups. However, there's been a noticeable increase in the number of people aged 30 to 79 diagnosed with hypertension. This underscores the need for timely health screenings and interventions to prevent the condition from getting worse [19]. Recent data from Canada's official health statistics indicate that individuals with type 2 diabetes consistently fail to meet the prescribed targets for hemoglobin A1C, low-density lipoprotein cholesterol, and systolic blood pressure [13]. Regular physical activity is associated with reduced sedentary time and improved overall fitness, which supports progress toward meeting physical health goals. Extended periods of sitting have been identified as a modifiable factor that notably contributes to economic challenges and health issues, such as diminished physical fitness. This underscores the necessity of evaluating strategies aimed at reducing cardiovascular risks through both diagnostic and therapeutic measures [5]. Physicians routinely perform evaluations, including the measurement of blood pressure, cholesterol, and glucose levels, along with prescribing medications that reduce the risk of conditions related to cardiovascular health and diabetes. This is implemented as a component of proactive care strategies instead of simply waiting for symptoms to manifest. Obesity not only strains cardiovascular function but also worsens insulin resistance and dyslipidemia [3,4]. These lifestyle factors are more prevalent in lower-income neighborhoods, where access to affordable, healthy foods and safe spaces for exercise is often limited. Promoting small, consistent changes such as walking instead of driving, choosing balanced meals, and maintaining a healthy weight can substantially improve both metabolic and cardiovascular health outcomes.

To address stagnant control rates, system-level interventions are needed. Community and primary care-based strategies using standardized treat-to-target algorithms, team-based care, fixed-dose combination therapy, and performance registries have shown measurable success in improving medication adherence and reducing cardiovascular risk [3,12]. Given that the CHMS summary tables provide only high-level population indicators, continued monitoring of prevalence, awareness, treatment, and control measures is essential for understanding changes in cardiometabolic health over time. Improved access to detailed, survey-weighted CHMS data would enable more precise evaluation of these indicators and allow future research to examine potential drivers of the observed descriptive patterns.

Taken together, these findings support the view that while hypertension management in Canada has benefited from structured guideline implementation and scalable treatment protocols, diabetes continues to present greater challenges due to its behavioral complexity and dependence on self-care. Integrating chronic-disease management linking primary care, community programs, and public-health surveillance can help bridge these gaps. Continued emphasis on patient education, equitable access, and culturally appropriate interventions remains essential for improving cardiometabolic outcomes nationwide.

Strengths and limitations

A key strength of this analysis lies in its use of nationally representative data from multiple CHMS cycles, enabling a comprehensive assessment of temporal patterns over more than a decade. The use of standardized measures and consistent survey methodology enhances comparability and reliability across time periods. Additionally, the application of weighted proportions ensures national representativeness of the findings.

However, since this study relied on aggregated CHMS summary tables, key limitations include the inability to analyze individual-level variables, apply survey-weighted methods, or assess determinants of awareness, treatment, and control. Future research using CHMS microdata with access to bootstrap weights would allow for more robust estimation of variance, testing of differences across cycles, and examination of factors associated with cardiometabolic outcomes. Such analyses could clarify whether the descriptive differences observed in the summary tables represent true population changes or sampling variability.

Given that diabetes indicators continue to rise and show limited improvement in control compared with hypertension, enhancing the capacity of primary care to deliver patient-centered and coordinated management will be essential to achieving better glycemic outcomes. Equally important is the emphasis on lifestyle modification encouraging physical activity, a healthy diet, adequate sleep, and reduced sedentary behavior [23]. These interventions should be coupled with culturally sensitive, community-based initiatives that reflect Canada’s diverse population.

Reducing socioeconomic inequalities, improving food security, and ensuring consistent access to healthcare services are also critical to narrowing gaps in cardiometabolic outcomes [24-26]. Strengthening national surveillance systems such as the CHMS, along with other data sources, will allow for ongoing evaluation of emerging challenges and guide the development of timely, targeted interventions.

In summary, these recommendations underscore the need for an integrated, interdisciplinary, and equity-driven approach to sustain long-term improvements in cardiometabolic health and to reduce the burden of hypertension and diabetes across Canada [27].

## Conclusions

Based on descriptive estimates from aggregated CHMS data, hypertension indicators among Canadian adults showed relatively stable patterns across the 2008-2019 survey cycles, with small fluctuations in awareness, treatment, and control that cannot be interpreted as statistically meaningful trends. Diabetes indicators suggested a gradual increase in the number of adults living with the condition over the same period, while estimates of glycemic control exhibited variability across cycles. Because the aggregated CHMS tables do not provide variance estimates, confidence intervals, or age-standardized values, these observations should be interpreted as descriptive patterns rather than confirmed temporal changes.

These findings highlight the utility of CHMS summary data for providing population-level snapshots of cardiometabolic indicators, while also emphasizing the need for future analyses using microdata and survey weights to conduct rigorous trend assessments. Continued monitoring of hypertension and diabetes through robust national surveillance systems remains important for informing prevention and management strategies in Canada.
